# Unusual Presentation of Hydatidosis - Neck Lump Causing Costo-Vertebral Erosion

**Published:** 2016-09

**Authors:** Mehtab Alam, Syed-Abrar Hasan, Shahab-Farkhund Hashmi

**Affiliations:** 1 *Department of Otorhinolaryngology, J.N.Medical College, A.M.U, Aligarh, INDIA.*

**Keywords:** Bone erosion, Primary hydatid cyst, Supraclavicular swelling

## Abstract

**Introduction:**

Hydatid disease caused by larval stage of Echinococcus has been recognized endemically in many countries. Liver and lungs are the most commonly affected organs. Involvement of the head and neck region is rare and bony erosion due to hydatidosis is even rarer.

**Case Report:**

We report a case of a 17-year-old girl from a poor socio-economic background who presented with a right sided supraclavicular lump, which after surgical excision and histopathological examination was diagnosed as hydatid cyst of neck.

**Conclusion:**

Because of its rarity in the neck region, primary diagnosis of hydatid cyst is overlooked and usually not included in the differential diagnosis of cystic neck swellings. A high index of suspicion is necessary to diagnose hydatid disease in an unusual location even in endemic areas.

## Introduction

Hydatid disease, also known as hydatidosis and cystic echinococcosis, is a cyclozoonotic infection caused by cestodes of genus echinococcus, usually Echinococcus granulosus (1). The endemic regions of human cystic hydatidosis are cattle and sheep rearing areas of South America, The Mediterranean region, middle east, southern and central Russia, India, and many parts of China (2,3).

For the completion of its life cycle, Echinococcus requires two distinct mamma- lian hosts. Dogs, wolves, and foxes are the definitive host. Intermediate hosts are herbivores like sheep and cattle (4). Humans are accidental intermediate hosts, which are infected by the oral ingestion of tapeworm eggs with contaminated food or water or by direct contact with the host (5).

Though hydatidosis can affect almost every part of the body, the liver and lungs are the most commonly affected organs. Multiple organ involvement is common. Primary hydatid disease involving the head and neck region is extremely rare even in geographical areas known to be endemic for echinococcus infestation. Until now, only a few cases of hydatid cyst have been reported in the head and neck, an unusual location (3,6). Among all the reported cases, bony erosion due to hydatid cyst is very rare in this region. In our case there was erosion of the transverse process of T1 vertebra along with erosion of the first rib, making this case one of the rarest of its kind in the head and neck region. Sureka J et al. reported a case of posterior skull base hydatid cyst causing destruction of the occipital and adjacent part of mastoid bones (7).

## Case Report

**Fig 1:**
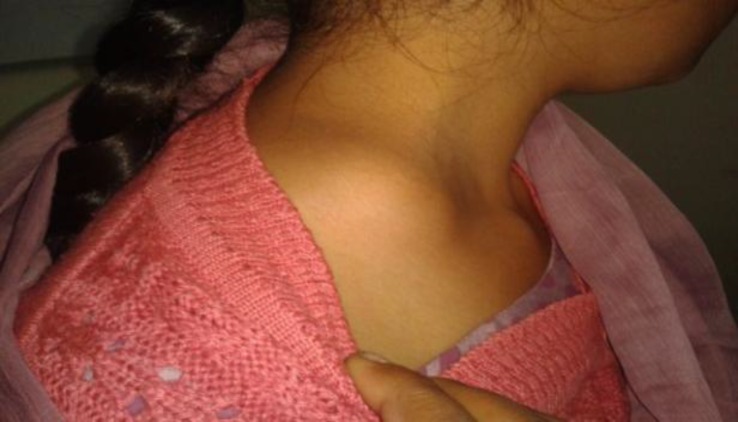
Swelling in right supraclavicular region without any signs of inflammation

A 17-year-old uneducated girl from poor socio-economic background residing in an overcrowded locality came to our OPD with a complaint of a lump on the right side of the neck in the supraclavicular region. She first noticed the swelling 4 months ago, which gradually increased in size without any pain. The detailed family history revealed that her mother suffered a case of pulmonary tuberculosis and was on anti-tubercular treatment. The patient had only been involved in household work without any engagement with pets. She was on mixed diet with normal appetite without any history of weight loss or fever. Clinical examination of the lump revealed a uniform non-tender, cystic swelling of around 5-6 cm with slight fluctuation as well in the right supraclavicular region (Fig.1). 

There were no signs of erythema and echymosis. A presumptive diagnosis of tubercular cold abscess was made on the basis of her family history. Complete blood count was normal except for slight eosinophilia. Mantoux test was negative and chest radiograph was absolutely normal. Absolute eosinophil count was raised (850 cells/microlitre). 

Ultrasonography of the neck showed a thick walled cystic lesion having internal septations and echoes with peripheral vascularity in the subcutaneous plane in the right supraclavicular region. Due to the USG findings, aspiration of the swelling was performed under aseptic conditions using an 18G needle. The aspirate in the syringe was clear watery fluid and was sent for cyto-chemical analysis. The fluid was reported to be acellular with some proteinaceous content. The swelling completely regressed after aspiration. Because of the clear fluid aspirate, the differential diagnosis of hydatid cyst was considered. Ultrasonography of the abdomen revealed no abnormality.

**Fig 2:**
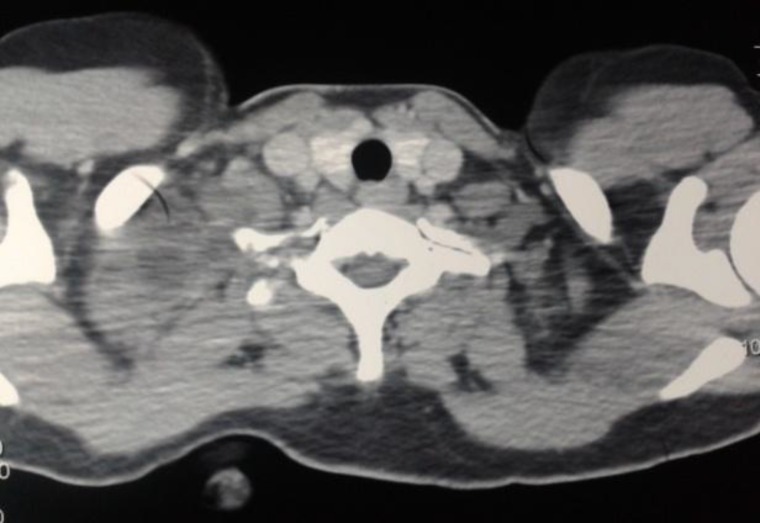
Contrast Enhanced CT image showing erosion of transverse process of T1 vertebra and 1st rib with maintained fat planes, by heterogenous enhanced lesion

On the 5^th^ post aspiration day, the patient presented with a tense and tender swelling in the same region. Again aspiration was done and pus came out in the syringe. Despite all our aseptic precautions, the swelling got infected during the first aspiration. Intravenous antibiotics were initiated and contrast enhanced computed tomography (CECT) of the neck and thorax was planned. CECT showed a heterogen- eously enhancing lesion at the level of the thoracic inlet just posterior to the lateral aspect of the right clavicle. Medially the lesion was abutting and causing erosion of the transverse process of T1 vertebra and the first rib (Fig.2). 

**Fig 3:**
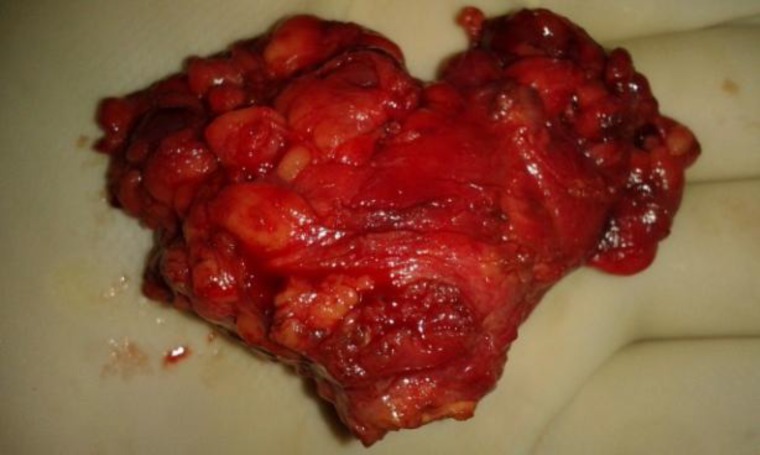
Surgically resected thick walled cystic lump

Posteriorly the lesion was abutting the scapula and subscapularis muscle with maintained fat planes. Overall CECT showed an infected nodal mass or complex cystic lesion. Bilateral lung fields were clear on CT. Surgical excision of the cyst was planned under general anaesthesia using supraclavicular skin crease incision. Surgical exposure revealed a thick walled cystic mass with numerous small lymph nodes and areas of fibrosis around it. The entirety of the mass was excised and sent for histopathology (Fig.3). Since we were keeping hydatid cyst as one of the our probable diagnosis, the wound was irrigated with hypertonic saline (20%) and betadine. Closure was done in three layers.

**Fig 4:**
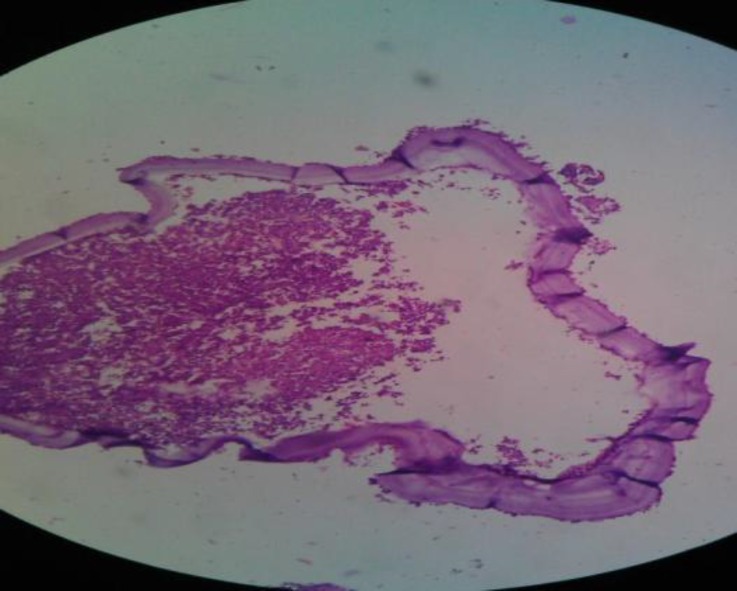
Histopathological examination revealing classical features of hydatid cyst

Histopathology confirmed the diagnosis for hydatid cyst by showing the presence of three classical layers: the innermost germinal layer, middle laminar membrane, and outermost thick pericyst (Fig. 4). During the post-operative period, Albendazole 400mg/day was given for 2 months and the patient was regularly followed up without any signs of recurrence.

## Discussion

Hydatid disease is a parasitic infestation which can involve any part of the body from head to toe (8).There are reports mentioning hydatid disease involving unusual sites like the spleen, kidney, heart, bone, and cranium but involvement of soft tissue comprises less than 3% of all hydatidosis (9). In the largest published series until now, only 24 out of 1056 cases of hydatidosis (only 2.3%) were localized in the soft tissue (10). It has been hypothesized that the presence of lactic acid in muscles does not allow the larvae to grow into cysts. Hydatid disease in the head and neck region is rare and only a few cases have been reported in literature (3). Rarity of the disease in this region presents a diagnostic dilemma for clinicians. Hidatid cyst is rarely considered in the differential diagnosis of cystic lesions in the head and neck especially in non-endemic areas in absence of hydatid disease elsewhere in the body. Multi organ involvement is seen in 20% to 30% of cases with liver involvement seen in all cases (11).

Among the few reported cases, our case is unique as there was bony erosion of the transverse process of T1 vertebra and the first rib noted on CT scan, making it probably the first reported case of cost-vertebral erosion due to hydatid cyst of neck.

Humans can serve as an intermediate host to echinococcal infection and acquire the infection either by direct contact with a definitive host like a dog or by ingestion of food or fluid contaminated with the ova of E.granulosus (12). Thus in a patient suspected of having hydatidosis, detailed inquiry should be made regarding occupation, residence, and family history. Our patient was a young girl who had not associated with pets or cattle and whose family had not been affected by hydatid disease.

The ova enters and hatch as embryos in the human small intestine and pass into the portal system or lymphatic system to reach the liver and lungs. Most of the larvae settles there to form hydatid cyst lesions. They can also cross the hepatic sinusoids or pulmonary capillary barrier and enter the systemic circulation, therefore affecting any part of the body (13). Hence there can be multi organ involvement in 20% to 30% cases. In our case the neck was the only region involved without any involvement of the liver or lungs.The nature of the signs and symptoms produced by hydatid cyst are extremely variable and never pathogonomic (14,15). In this patient the only presenting symptom was progressively increasing painless swelling in the right supraclavicular region.

The diagnosis of hydatid disease mainly depends on clinical history, radiological imaging, and serological tests. Imaging techniques like CT, USG, and MRI remain more sensitive than serodiagnosis. Even in the presence of negative serologial results, a characteristic radiological demonstration should still point to the diagnosis of echinococcus (16).

These help to determine the cystic avascular nature of the lesion. Within the lesion internal septations, daughter cysts, and vesicles can also be seen (8,14).

In this patient, CECT of the neck and thorax did not show any of the mentioned findings as it was done after first fine needle aspiration following which the lump got infected. One peculiar finding on CT was costo-vertebral bony erosion. USG showed internal septations without any characteristic hydatid sand or daughter cysts.

Although our patient did not undergo any serological procedures, tests like hemagglutination, latex agglutination, skin test (Casoni intradermal test ), ELISA, and western blot are widely used especially in abdominal disease as they have low diagnostic sensitivity and specificity in extra hepatic hydatidosis (4). These tests are important in follow up of treated patients as post-operative raised titres points towards recurrence (17). The role of FNAC in hydatid cyst is controversial and is usually not favored because there is the possibility of anaphylactic reaction, dissemination of the disease, and recurrence as a result of spillage of contents (18,19).

We performed fine needle aspiration as hydatidosis was not considered as the differential diagnosis and, when the aspirate yielded clear fluid, only then did we think of echinococcal infection as one of our probable diagnosis. Luckily there was no anaphylaxis though the mass got infected following aspiration.

The most effective treatment for hydatid cyst is surgical removal and utmost care should be taken to prevent rupture of the cyst as spillage of the contents can result in anaphylactic reaction, recurrence, and multiple hydatidosis (6). If the definitive diagnosis of hydatid cyst has been made pre-operatively then preliminary aspiration and instillation of hypertonic saline (20%), silver nitrate (0.5%), and formalin can be used to prevent seeding of the cyst contents and to inactivate the protoscolices (3). In this case as the definite diagnosis of hydatid disease was not made pre-operatively and also because the mass got infected on initial aspiration, instillation of any scolocidal was not tried. Though the cyst was completely removed without any rupture, the wound was irrigated with hypertonic saline and povidine-iodine solution. The surgical specimen was sent for histopathological examination, which revealed characteristic features of hydatid cyst. Albendazole (400mg/day) was started postoperatively and continued for 2 months and the patient did not show any signs of recurrence in follow up.

## Conclusion

Even in an endemic area a high degree of suspicion is required for diagnosing hydatid disease in an unusual location like the neck, where they may lead to bony erosion. Thus it should be considered in the differential diagnosis of slow growing cystic swellings of the neck. Radiological imaging facilitates diagnostic and therapeutic procedures in view of complete surgical removal, following which the prognosis is excellent.
